# Feasible Influence of G-CSF on Clinical Pregnancy Outcome in Oocyte Donation Cycles for Patients with Recurrent Implantation Failure

**DOI:** 10.3390/medicina60060966

**Published:** 2024-06-11

**Authors:** Nataliya Kushniruk, Anna Stastna, Tomas Fait, Tereza Lenertova

**Affiliations:** 1 1st Faculty of Medicine, Charles University Prague, 120 00 Prague, Czech Republic; nata.ku08@gmail.com; 2 Department of Demography and Geodemography, Faculty of Science, Charles University Prague, 120 00 Prague, Czech Republic; 3 Department of Obstetrics and Gynecology, 2nd Faculty of Medicine, Charles University Prague, 150 00 Prague, Czech Republic; 4 Department of Health Studies, Polytechnic College Jihlava, 586 01 Jihlava, Czech Republic; 5 FertiCarePrague, SE, 150 00 Prague, Czech Republic

**Keywords:** implantation failure, intrauterine lavage of G-CSF, oocyte donation cycle, pregnancy rate, endometrial receptivity

## Abstract

*Background and Objectives:* The aim of our single-center cohort study was the determination of the influence of the intrauterine lavage of granulocyte colony-stimulating growth factor (G-CSF) on clinical pregnancy rate in patients with a history of implantation failure older than 40 years. *Materials and Methods:* The study was conducted in Ferticare Prague SE between May 2018 and June 2020. Overall, 115 patients were distributed into two arms, with 48 subjects in the experimental and 67 in the control arm. All women have had a previous history of unsuccessful history of infertility treatment with their own genetic material and at least one ineffective cycle with the donated oocytes. The experimental arm underwent the intrauterine lavage of 0.5 mL of pure G-CSF from 120 to 48 h prior to embryo transfer. *Results:* The clinical pregnancy rate was 63.3% in the experimental arm and 47.8% in the control arm (*p* = 0.097 for Pearsonߣs χ^2^, and *p* = 0.133 for Fisher’s exact test). However, the mean endometrial thickness on the day of embryo transfer did not appear to be statistically different (*p* = 0.139). Only the difference in endometrium thickness growth was statistically significant (*p* = 0.023). The increase in pregnancy rate is still encouraging for the future, even if it is not significant. *Conclusion:* Our study suggests the trend of increased pregnancy rate after the intrauterine G-CSF lavage in the interval of 120–48 h prior to embryo transfer.

## 1. Introduction

A recent trend in reproduction in developed countries is fertility postponement [1]. The increased number of patients over forty years who want to be pregnant is the main reason for the wide popularity of oocyte donation (OD) programs due to the low ovarian reserve and poor quality of oocytes [2]. Oocyte donation programs may neglect or maximally limit implantation failures; however, they still exist [3].

According to the information of the National Center for Chronic Diseases Prevention and Health Promotion in the USA, in 2016, the percentage of OD cycles grew from 10% for women at the age of 42 to 60% for women at the age of 48 [4].

Despite the fact that OD is the most successful assisted reproduction technique program, with a pregnancy rate of about 65% and a live birth rate of about 55% [5], there is still a subgroup of patients with implantation failure.

Recently, many scientific publications have addressed the program of recurrent implantation failure among in vitro fertilization patients who use their own genetic material, and it was defined as the failure of implantation for at least three cycles with a total number of four good quality embryos for the patients under 40 years [6,7,8]. Pirtea [9] defined recurrent implantation failure as a rare condition, with only less than 5% of euploid blastocysts in patients under 40 years. These patients would fail to achieve pregnancy with three embryos transferred. Moreover, clinically recognized pregnancy loss occurs in approximately 15–20% of pregnancies; meanwhile, recurrent pregnancy loss, defined as two or more failed clinical pregnancies consecutively, occurs less than in 5% of cases, and only 1% experience three or more.

There is no clear definition of recurrent implantation failure for the OD cycles, especially in the category of patients in the advanced reproductive age. This above-mentioned category of patients deserves our interest as a result of delayed childbearing. 

It is difficult to originate the cause of implantation failure because possible causes may be predetermined by embryo quality [8] or endometrial quality along with the intrauterine environment [10,11].

It seems to be logical to check the euploidy of embryos with pre-implantation genetic testing (PGT) prior to the transfer; however, the high incidence of mosaicisms and false negative results make this method increasingly controversial [12]. Tests performed on day 3 and day 5 for the same embryo detect the decreased number of euploid embryos within the group of the increased female age [13]. The chromosome imbalance and mosaicism still may be the cause of failed transfers of euploid embryos [14].

In the study, we have selected patients with artificial hormonal supplementation for both follicular and luteal menstrual phases because of the prevention of a decrease in HOX gene expression for the safety of endometrial receptivity [15]. We know that the intrauterine environment and the endometrial–embryonic interaction at the implantation period depends on the expression of Homeobox genes along with integrin β_3_, IGFBP-1 (insulin-like growth factor binding protein-1), FOXO (fork-headed box proteins) transcription factors, osteoponin expression, interleukins, TNFα, interferon β, mucin, macrophage migration inhibitory factor, exotoxin, VEGF (vascular endothelial growth factor). It is the reason for appropriate hormonal maintenance of both phases of the menstrual cycle [16,17,18].

That was the predisposition of the current study—identifying the feasible influence of granulocyte colony-stimulating factor (G-CSF) on pregnancy rate by means of improving endometrial receptivity [5,19,20,21,22,23].

## 2. Materials and Methods

**Figure 1:**
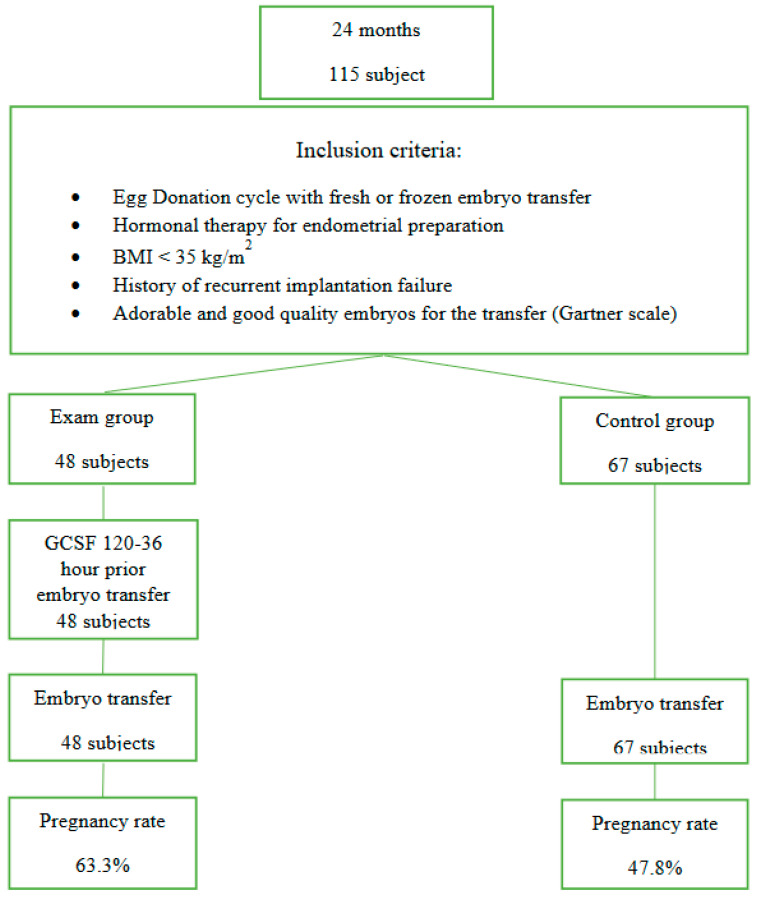
Study design. BMI (body mass index).

The single-center study was held for a period of 24 months at the Ferticare Prague clinic from May 2018 to June 2020. Inclusion criteria were the recurrent implantation failure with own oocyte (minimally two) and minimally one implantation failure with donated oocytes, age over 40 years, and donated oocyte cycle. The cohort of 115 nulliparous women was randomized into two arms: the experimental (n = 48) and the control (n = 67). Both arms included just oocyte donation cycles, no matter whether fresh or frozen embryos have been transferred (Figure 1). Women with body mass index (BMI) over 35 kg/m^2^ and with known factors for implantation failure (immunologic factors, inborn defects of the uterus, thrombophilia) were excluded from the study. For both groups, only excellent, good, and normal-quality embryos (*Gartner scale*) were enrolled. The laboratory conditions remain equal for all embryos.

Both the control and the exam arms were similar in age and BMI. The mean age was 42.03 and 42.2 years respectively. There are no significant biases by age within both arms (*p*-value 0.838). Mean BMI was 23.65 ±3.27 kg/m^2^ in the exam and 23.01 ±3.44 kg/m^2^ in the control arm (*p*-value is 0.315) (Table 1). No difference was found in the personal history of birth, and all women were nulliparous. The age of donors was 25 to 32 years for both arms. We did not control smoking status, but all women were strictly recommended not to smoke.

We have performed the endometrial measurement between the 10th and the 13th day of endometrial preparation by vaginal ultrasound. The endometrial growth was supported by hormonal therapy—estradiol for the proliferative phase and progestins for the luteal phase. The mean oral daily dose of estradiol was 7.4 ± 1.97 mg in the experimental arm and 8.0 ± 2.30 mg in the control arm (*p*-value 0.147). We generally used 600–800 mg of micronized progesterone vaginally daily in combination with injected progestin (progesterone oil solution for intramuscular injections 30 mg in mL, 2 mL) once a week. 

Subjects from the experimental arm had additional intrauterine lavage of G-CSF 30 MU/0.5 mL (Zarzio, Sandoz GmbH, Austria) in the period of zero up to 72 h of progesterone administration. This means 120 to 36 h before the embryo transfer. It was applied through the Wallace Classic Embryo Transfer Catheter. 

Statistical analysis was performed with the help of IBM SPSS Statistics 28 (IBM, New York, NY, USA). The main indicators analysed in the paper are endometrial thickness, endometrial thickness growth, and clinical pregnancy rate. We have applied the mean ± standard deviation (SD), and differences between groups were calculated with the two-tailed Student’s *t*-test for unpaired data, two-tailed Student’s *t*-test for paired data, and was controlled by a non-parametric Mann–Whitney test and Wilcoxon signed rank test in cases where the normality of distribution was violated. Moreover, the *χ*^2^ test was implied. *p* < 0.05 was considered statistically significant.

## 3. Results

The difference in endometrium thickness between the experimental and control arms on days 10–13 of endometrial preparation was not significant (7.69 ± 1.40 mm for the experimental arm and 7.70 ± 1.32 mm for the control arm, *p* = 0.139). Between days 10–13 of endometrial preparation and the day of embryo transfer, both experimental and control arms experienced a significant increase in endometrial thickness, with a mean growth of 1.122 mm for the experimental arm and 0.739 for the control arm. The growth of endometrial thickness was significantly higher in the experimental group (*p* = 0.023) (Table 2). 

In both arms, a similar number of embryos were transferred onto 125–128 h of embryo culture, corresponding to 120–128 h of progesterone administration; the mean number of embryos transferred is 1.73 ± 0.45 in the experimental arm and 1.72 ± 0.45 in the control arm (*p* = 0.905). 

The clinical pregnancy rate (PR) was 63.3% in the experimental arm and 47.8% in the control arm. Although these results indicate an important difference in pregnancy rates, these differences are not significant at the chosen level (*p* = 0.097 for Pearsonߣs χ^2^ and *p* = 0.133 for Fisher’s exact test), mainly because of the size of the observed cohort of women. 

## 4. Discussion

This was the key point of our research, try to find the practical supporter on the way of solving implantation failure for patients with the oocyte donation cycle. In our study, we rely on the young age of the egg donors and their proven euploidy and the absence of severe sperm deviations, and thus, we exclude embryo quality as the principal cause of implantation failure. This enables us to concentrate on the endometrial quality.

The recombinant G-CSF is widely used for preventing neutropenia-related infections and mobilizing hematopoietic stem cells [24].

G-CSF was used in culture media with statically nonsignificant growth of pregnancy rate [25].

Meta-analysis of Xie aimed to explore the efficiency of intrauterine perfusion of G-CSF on infertile women with thin endometrium. Eleven eligible studies involving 683 patients were included in this meta-analysis. G-CSF perfusion could significantly improve endometrial thickness (mean difference 1.79, 95%CI: 0.92–2.67) and clinical pregnancy rate (RR 2.52, 95% CI 1.39–4.55) [26].

Intrauterine G-CSF administration showed maximal effects 24 h after administration in enhancing endometrial receptivity and subsequent increase of angiogenesis by demonstrating elevated integrinβ3 and OPN and reduced cytotoxicity of NK cells. It promoted the stability of attached embryos at the early stage of implantation in vitro [2].

Recently, several studies were performed regarding the impact of G-CSF on the pregnancy rate of patients with recurrent implantation failure in different age groups among the patients with their own genetic material. Studies by Davari-Tanha et al. and Hou et al. have shown the positive impact of G-CSF on the pregnancy rate in both fresh and frozen cycles for in vitro fertilization (IVF) patients under the age of 40 years [19,27].

On the contrary, Kalem et al. did not observe the positive impact of the G-CSF on the pregnancy rate of patients with recurrent implantation failure in a similar age group [20]. Kamath et al. have performed the collected data on 13 trials in the Cochrane database with remaining uncertain results on the improvement in clinical pregnancy rate, while some of the trials showed the improvement in pregnancy rate; otherwise, they were of low-quality evidence [21].

Also, the recent ESHRE recommendation on recurrent implantation failure does not recommend G-CSF intrauterine lavage because of the conflicting evidence [28].

We have chosen OD cycles due to the fact that presumably good-quality oocytes minimize the potential impact of oocytes or embryo quality. We did not perform PGT-a (pre-implantation genetic testing for aneuploidy) because of significant trophectoderm mosaicisms and, thus, a potentially plausible percentage of false aneuploidy embryos [12,29]. 

The priority of our study is the age of women over 40 years and only oocyte donated cycles. The majority of studies were concentrated on the category of patients with their own genetic material, predominantly in the age group under 40 years. However, we targeted oocyte donation cycles in both groups of patients, mainly for similar endometrial conditions. We tried to optimize the secretory endometrial phase by artificial progesterone support [22,30]. We presume the formation of appropriate superficial stromal edema, leading to endometrial thickening, by measurement of endometrial thickness on the day prior to progestin and on the day of embryo transfer [17]. Moreover, it was mentioned the positive impact of endometrial injury upon the implantation rate; thus, we may predict the slight intrauterine intervention by means of lavage leading to intrauterine environment changes, thus providing a significant increase in pregnancy rate between experimental and the control groups in our study [15]. Data are indicative of the impact of G-CSF on neoangiogenesis by changes in intrauterine concentrations of TGF-β, PDGF, IGF, VEGF, EGF, and FGF-2 [31,32,33].

Enciso et al. performed the analysis of the window of implantation and confirmed that the majority of patients are receptive between day 5 and day 6 of progesterone intake, and just in a few cases, the implantation window occurs early after 2.5 days up to 8 days of progesterone support [34]. 

Thus, the embryo transfer in our study was performed in the optimal interval of progesterone intake, which is why we disregard the possible bias in this presumably small group of patients.

## 5. Conclusions

Our study suggests the trend of increased pregnancy rate after the intrauterine G-CSF lavage in the interval of 120–48 h prior to embryo transfer. The growth of the endometrial thickness was statically higher after G-CSF intrauterine lavage. 

We suggest using G-CSF lavage in the cycles with complex endometrial preparation for the category of patients in advanced reproductive age and recurrent implantation failure. It could induce changes in the intrauterine environment and increase the endometrial receptivity.

## Figures and Tables

**Table 1:** Characteristics of study arms—age, BMI, number of embryos transferred.

Index	Experimental Arm	Control Arm	*p*-Value
Age	42.2 ± 4.92	42.03 ± 4.30	0.838
BMI	23.01 ± 3.44	23.65 ± 3.27	0.315
Number of embryos transferred	1.73 ± 0.45	1.72 ± 0.45	0.905

Data presented as mean ± SD, and differences between the groups were tested by a two-tailed Student’s *t*-test for unpaired data.

**Table 2:** Endometrial thickness (mm) in study arms.

Endometrial Thickness	Experimental Arm	Control Arm	*p*-Value *
Endometrium on G-CSF day	7.69 ± 1.40	7.70 ± 1.32	0.955
Endometrium ET day	8.81 ± 1.44	8.44 ± 1.24	0.139
Mean endometrial growth	1.122	0.739	0.023
Difference between beginning and end (*t*-test for paired data)	*p* < 0.001	*p* < 0.001	

Data presented as mean ± SD, * differences between the groups were tested by two-tailed Student’s *t*-test for unpaired data.

## Data Availability

Data is unavailable due to ethical restrictions.
